# Expanding perspectives on specialized metabolism in *Streptomyces* bacteria

**DOI:** 10.1042/EBC20250045

**Published:** 2026-08-19

**Authors:** Lauren A. Tiller, Katharine P. McCoy Bridges, Hindra, Marie A. Elliot

**Affiliations:** Department of Biology, Michael G. DeGroote Institute for Infectious Disease Research, McMaster University, 1280 Main Street West, Hamilton, ON, L8S 4K1, Canada

**Keywords:** cell type, regulation, specialized metabolism, Streptomyces, trafficking

## Abstract

*Streptomyces* bacteria are prodigious producers of specialized metabolites, although when, why, and how they make many of these molecules is a puzzle that scientists have been working to solve for decades. While impressive progress has been made, new paradigms continue to be revealed, and new discoveries are increasingly facilitating access to the untapped metabolic potential of these bacteria. Here, we summarize recent advances in the specialized metabolism field that expand our views of what is possible, from regulation, production, and dissemination perspectives. We discuss the interplay between cluster-situated regulators and genes outside their biosynthetic clusters, as well as the intersection of these ‘base regulators’ with more globally acting pleiotropic regulators and nucleoid-associated proteins. We further describe the competition between different pleiotropic regulators and how this collectively enables the efficient integration of multiple regulatory inputs into biosynthetic gene cluster control. Finally, we highlight the production potential of cell types and growth stages that have not historically been associated with specialized metabolism, and describe intriguing containment strategies for biosynthetic enzymes and for the distribution of their metabolic products.

## Introduction to *Streptomyces* and specialized metabolites

**Figure 1:**
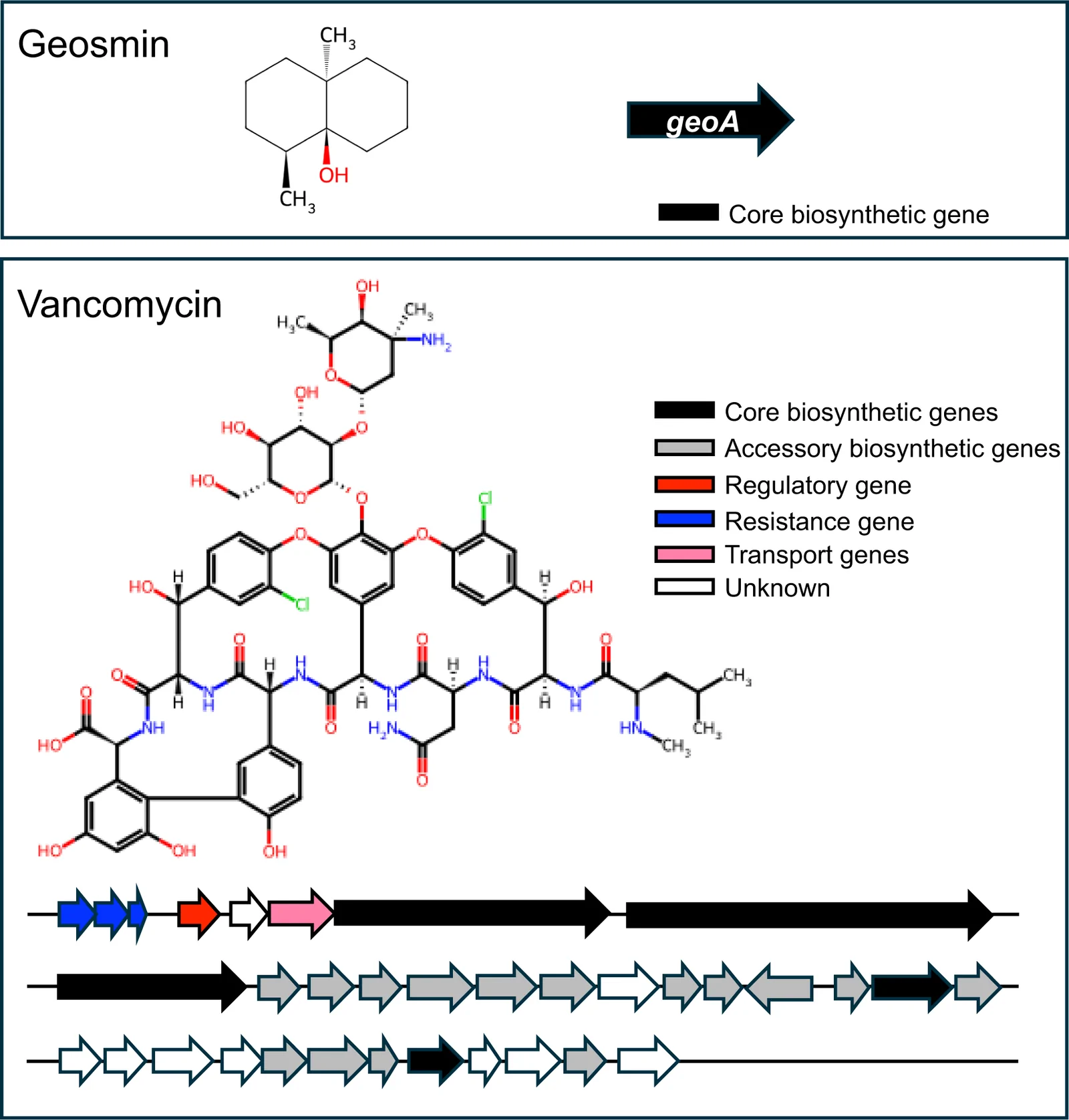
Comparison of simple and complex specialized metabolites and their associated BGCs **Top panel:** Structure of the volatile terpene geosmin and its (single) biosynthetic gene. **Bottom panel:** Structure of the glycopeptide antibiotic vancomycin and its associated BGC. Predicted/established functions for the different gene products are indicated in color as per the legend. Geosmin and vancomyin structures were sourced from ChemSpider.

Specialized metabolites are remarkably diverse organic molecules that are produced by a wide range of organisms, including many bacteria. Their synthesis is directed by the products of biosynthetic gene clusters (BGCs), with any given BGC typically containing all the genes needed to produce one specialized metabolite; these can include biosynthetic genes, regulators, transporters, and immunity genes (Figure 1). The resulting compounds encompass everything from simple molecules, like the small terpene geosmin that gives soil its earthy odor, through to extraordinarily complex molecules like the glycopeptide antibiotic vancomycin (Figure 1). These molecules are often produced under specific growth conditions and are presumed to confer their producing host with a competitive fitness advantage. Metabolically gifted bacteria are frequently found in the environment, where specialized metabolites enhance their survival in the face of dynamic physical conditions, changing nutrient availabilities, and the presence of diverse bacteria, fungi, protists, and higher-order organisms.

One of the richest sources of specialized metabolites are *Streptomyces* bacteria [1]. These microbes are ubiquitous in the soil, as well as in marine habitats, and any given *Streptomyces* species encodes a unique complement of ∼10–80 BGCs [2], with these numbers continually changing as new BGC families are identified [3]. Intriguingly, the products of the majority of BGCs have yet to be identified or characterized [4], due in part to the fact that most BGCs are expressed at low levels under conventional laboratory growth conditions.

As a complement to their complex chemical repertoire, *Streptomyces* also have a sophisticated developmental cycle that is highly responsive to environmental conditions [5]. Their classical life cycle initiates with the emergence of germ tubes from previously dormant spores. These vegetative cells grow by tip extension and branching to yield a dense network of filamentous cells known collectively as the vegetative mycelium. Environmental stresses such as nutrient limitation are thought to promote a switch from vegetative growth to reproductive growth, where non-branching aerial hyphae are erected above the vegetative mycelium. These aerial hyphae are then subdivided into pre-spore compartments, which go on to mature into chains of dormant spores. Following spore germination, a subset of *Streptomyces* species can embark upon an alternative developmental trajectory termed ‘exploration,’ in response to specific nutritional conditions. Exploration involves the rapid expansion of (largely) vegetative hyphae across the surface of solid media and invokes different growth strategies [6] and distinct genetic programs [7–9] relative to the classic developmental cycle.

There is little known about how specialized metabolism is governed during exploratory growth and where within the exploring colony it occurs. In contrast, specialized metabolism has been well-studied in the context of classical development, with the onset of reproductive growth coinciding with the initiation of specialized metabolite synthesis [10–14]. Notably, aerial development and specialized metabolism share some genetic elements (e.g., [14–18]), but are generally considered to be spatially segregated, with specialized metabolism largely being confined to a subset of vegetative cells and excluded from reproductive (sporulating) cells [19–21].

## Production of specialized metabolites by unconventional growth and cell types

**Figure 2:**
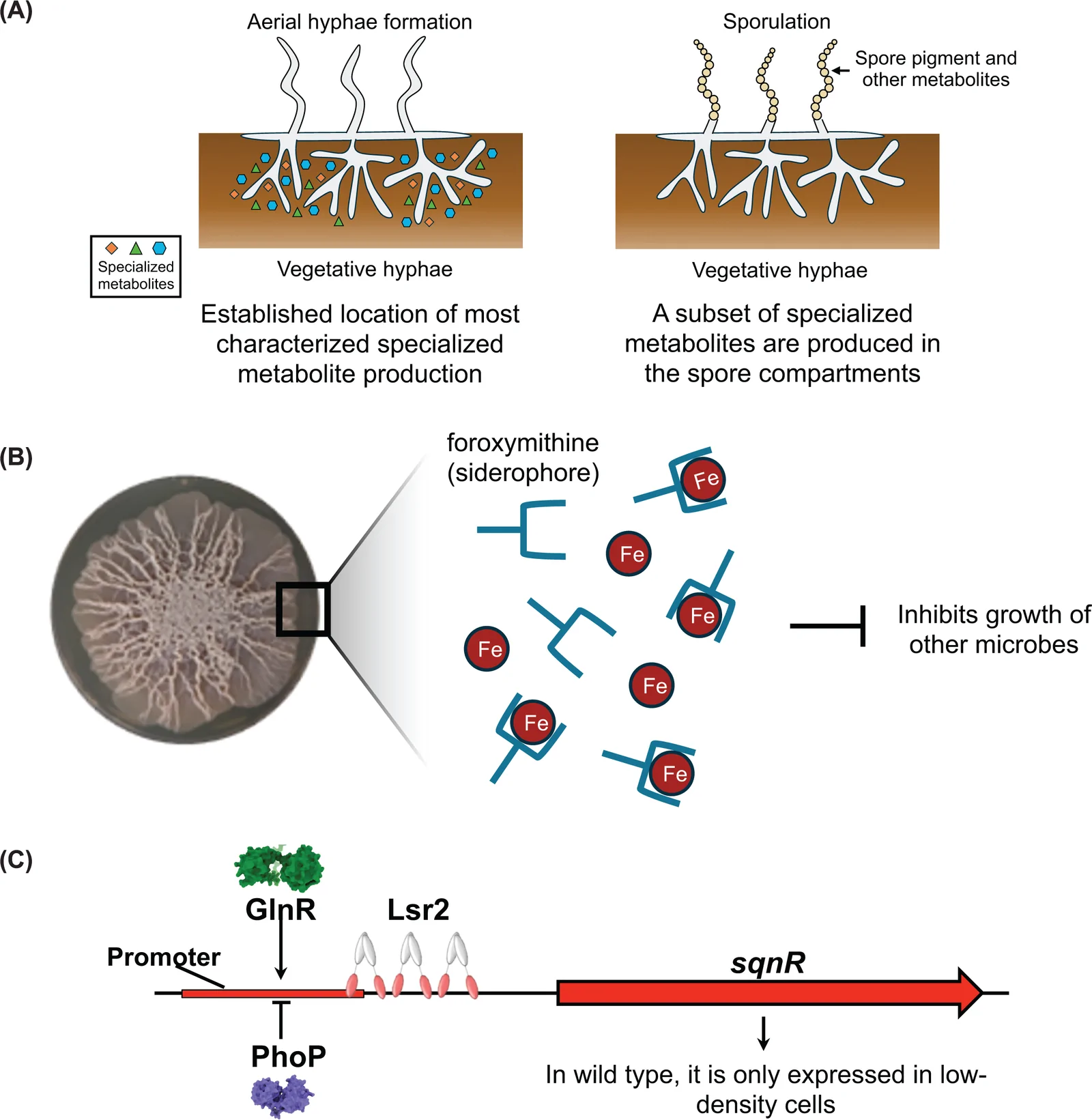
Specialized metabolite production in unusual cell types (**A**) *Left:* Specialized metabolite production is typically thought to occur within a subset of vegetative hyphae at the same time that reproductive growth (aerial hyphae) formation begins. *Right:* During sporulation, a polyketide specialized metabolite is incorporated into the spore coat; there is additional evidence that other antibiotics can be contained and/or produced in the spores. (**B**) *Streptomyces venezuelae* exploration occurs in response to specific growth conditions. The addition of glycerol to conventional exploratory growth medium (yeast extract and peptone) promotes the release of the diffusible siderophore foroxymithine, which inhibits the growth of nearby bacteria and enhances colony fitness when grown in competition with other microbes. (**C**) *Streptomyces* species WAC07094 produces the potent antibiotic saquayamycin during low-density growth. This ‘anti-quorum’ behavior is controlled by the cluster-situated regulator SqnR, whose gene is under the control of multiple regulators, including the two-component regulator PhoP (repressor), the orphaned two-component regulator GlnR (activator), and the nucleoid-associated protein Lsr2 (repressor).

The division of labor between reproductive cells and specialized metabolite-producing vegetative cells is not absolute. One of the final stages of *Streptomyces* spore maturation involves the production of a pigmented polyketide metabolite that gets deposited into the spore coat, giving mature spores their distinct coloration (e.g., [22,23]). The spores of *Streptomyces griseus* have also been reported to contain the aminoglycoside antibiotic streptomycin [24] (Figure 2A). It was suggested that there could be an ecological benefit to having an antibiotic reservoir available during spore germination to minimize the growth of competitor bacteria and maximize colony establishment by the producing *Streptomyces* species. These observations suggest there is value to considering alternative cellular compartments as sites of specialized metabolism when screening for these natural products.

*Streptomyces* exploration provides an interesting example of a situation where new metabolites are produced in association with alternative growth modes. Growing *Streptomyces venezuelae* on a modified exploration-promoting medium (YPG, containing yeast extract, peptone and glycerol) led to the identification of an uncommon siderophore known as foroxymithine [25]. Foroxymithine is readily diffusible and effectively inhibits the growth of other microbes in the vicinity of the producing colony (Figure 2B). This siderophore is also critical for *S. venezuelae* fitness during growth in competitive environments, which was surprising given that exploring colonies produce other iron-chelating molecules [25,26]. This lack of siderophore redundancy suggests that *Streptomyces* have evolved creative ways of diversifying and differentiating specialized metabolites that share the same general functionality, and raises the interesting question of what conditions prompt the production of siderophores having different properties.

When considering conditions where specialized metabolites are being produced, growth density is another factor that is consistently associated with *Streptomyces* metabolic output. Increased cell density coincides with the accumulation of so-called ‘quorum-sensing’ signaling molecules, many of which are themselves specialized metabolites. In *Streptomyces* and related actinobacteria, the best-characterized signaling molecules are the γ-butyrolactones [27–29], with additional furan and butenolide compounds serving equivalent roles [30–32]. High concentrations of these signaling molecules can trigger antibiotic production, with positive correlations having been made between cell density and metabolite output for *Streptomyces clavuligerus*, in the form of cephamycin C and clavulanic acid production, as well as for *Streptomyces griseus* and its synthesis of streptomycin [33,34], amongst many others.

While laboratory-based studies have largely focused on metabolite production by high-density cultures, in many natural habitats, microbial populations are too sparse to trigger quorum (high-density)-dependent behaviors [35,36]. Indeed, studies are now revealing that low-density regulatory circuits can also promote specialized metabolism in some streptomycetes. The wild *Streptomyces* isolate WAC07094 produces the angucycline-like antibiotic saquayamycin during low-density growth, and this stems from the fact that its cluster-situated transcriptional regulatory gene *sqnR* is most highly expressed under conditions of low cell density [37]. *sqnR* is subject to multilevel transcriptional control, including (i) repression by the PhoPR two-component system, where the PhoP response regulator represses *sqnR* under conditions of high cell density; (ii) repression by the nucleoid-associated protein Lsr2; and (iii) activation by the nitrogen-responsive regulator GlnR [37] (Figure 2C and below for more detail on each of these regulators). It seems unlikely that this low-density antibiotic production is a unique situation, and it will be interesting to see how many uncharacterized BGCs and their associated molecules could be activated and detected, respectively, by expanding the cell types and culturing strategies (e.g., [38]) used to mine for new specialized metabolites.

## Control of BGCs and specialized metabolism

Decades of work have been devoted to understanding the regulation of BGCs within the streptomycetes, and a consistent theme is that these BGCs are subject to a multitude of regulatory inputs, as illustrated by the saquayamycin cluster control described above [37]. Many BGCs encode dedicated ‘cluster-situated regulators’ that can positively or negatively impact cluster expression, usually through direct binding to promoter regions within their cognate BGCs. The activity of these cluster-situated regulators can be tuned by environmental and cellular conditions (e.g., redox [39]), and the regulators themselves can be responsive to diverse ligands (reviewed previously in [40]). Their expression is also often subject to control by diverse ’pleiotropic regulators.’ These pleiotropic regulators typically have multiple targets on the chromosome that can include primary metabolism genes, developmental genes, and other BGCs. While significant progress has been made in understanding the contributions by both cluster-situated regulators and pleiotropic regulators to BGC control, there remain many gaps in our knowledge, with BGC control remaining a rich source of new and interesting regulatory circuitry.

**Figure 3:**
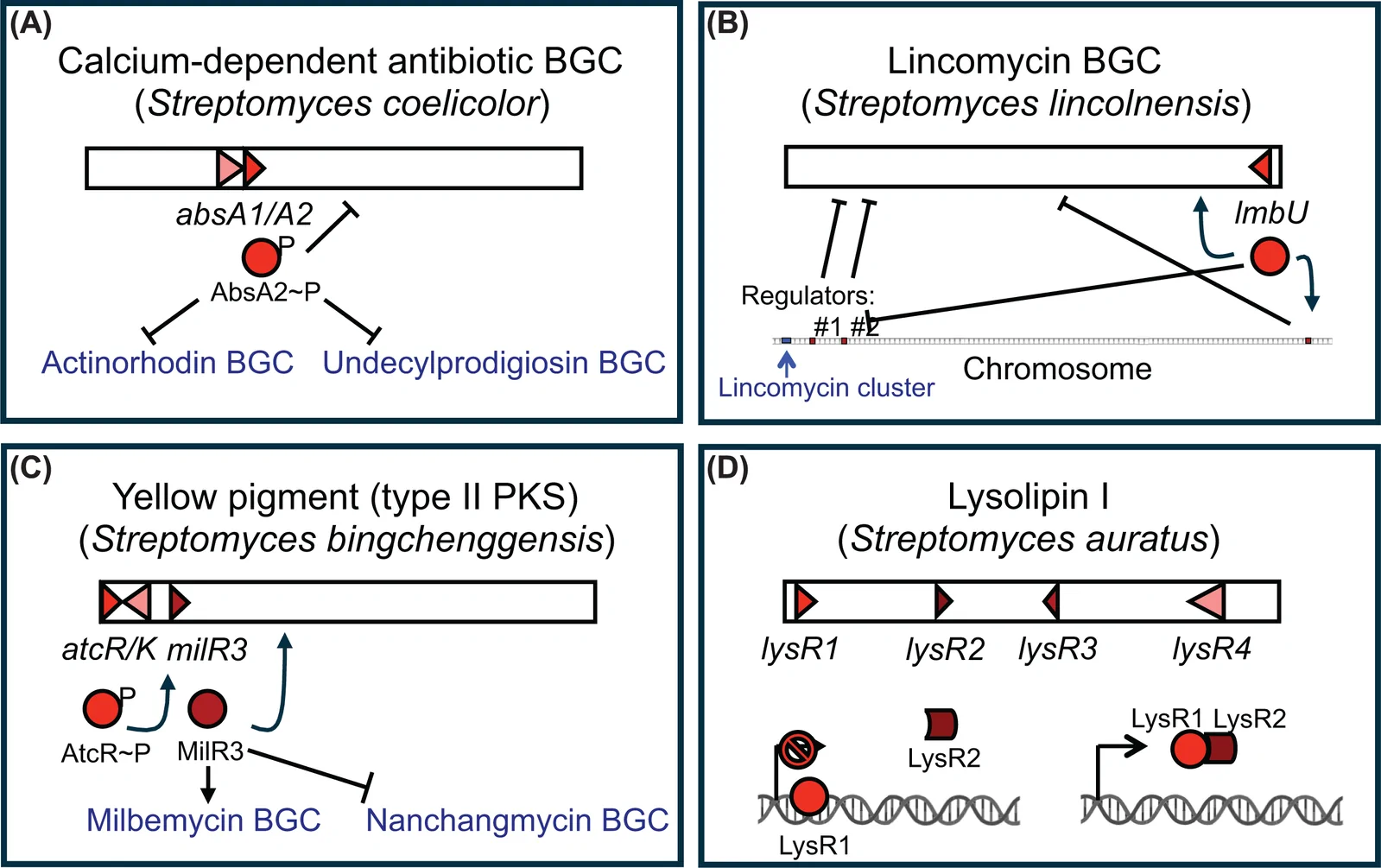
Regulatory interplay between cluster-situated regulators and their diverse target genes (**A**) Early example of the cluster-situated regulator (AbsA2, a two-component regulator that is phosphorylated by its associated histidine kinase AbsA1) for the CDA, controlling the expression of other BGCs in *Streptomyces coelicolor*. (**B**) The cluster-situated LmbU regulator in *Streptomyces lincolnensis* activates the expression of the lincomycin BGC and differentially affects the expression of three cluster-external regulators whose positions are indicated on the chromosome schematic at the bottom of the panel (relative to the lincomycin BGC that is indicated in blue, with the external regulators marked by the #'s 1-3). LmbU represses the first two regulatory genes (*slinc_*0469 and *slinc_*1037) and activates the third (*slinc_8097*). Each of these three external regulators in turn function to (directly or indirectly) repress lincomycin expression. (**C**) The unusually oriented two-component regulator-encoding *atcR* (response regulator) and *atcK* (histidine kinase) activate the expression of an additional regulator (*milR*) situated within the yellow pigment type II polyketide BGC. Arrows indicate activation; perpendicular lines indicate repression. MilR then activates the expression of both its own cluster and that of the milbemycin cluster, while also repressing the nanchangmycin BGC. (**D**) The lysolipin I cluster from *Streptomyces auratus* encodes four cluster-situated regulators. LysR1 functions to repress cluster expression, and its repression is alleviated through binding and sequestration by LysR2, leading to cluster activation.

The base regulatory unit of most BGCs is their cluster-situated regulator(s). These transcription factors were historically viewed as acting primarily within the context of their associated BGC. However, it is now clear that there is considerable regulatory interplay between cluster-situated regulators and other BGCs (e.g., [41–43]). The first demonstrated example of this came from early studies of the *absA1/A2* two-component regulatory system in the model system *Streptomyces coelicolor* [44]. The *absA* cluster was one of the first characterized pleiotropic regulators of antibiotic production, with its mutation leading to defects in the production of multiple antibiotics in *S. coelicolor* [45]. Subsequent mapping of its chromosomal location led to the discovery that the *absA1/A2* genes were embedded within the calcium-dependent antibiotic (CDA) BGC and, as such, would be considered a cluster-situated regulator [46]. Biochemical analyses revealed that phosphorylated AbsA2 directly controlled the expression of multiple BGCs (including the CDA cluster) by binding to the promoter regions of cluster-situated regulators [47] (Figure 3A).

In addition to controlling the expression of other BGCs, cluster-situated regulators can also govern the expression of ‘cluster-external’ regulators that function to fine-tune BGC expression. This has been seen for the regulator LmbU, which is both the founding member of a family of new antibiotic regulators and the cluster-situated activator of the lincomycin BGC [48,49] (Figure 3B). Subsequent work has revealed that LmbU also directly controls the expression of three regulator-encoding genes that are distributed across the chromosome (outside of the lincomycin cluster). LmbU represses the expression of two of these regulators and activates expression of the third. Interestingly, each of these LmbU-governed regulators contributes to repression of lincomycin biosynthesis [50], allowing for precise control of cluster expression (Figure 3B).

Studies into the regulation of specialized metabolite production are also revealing unusual genetic organizations and novel regulatory strategies. When probing the specialized metabolism of *Streptomyces bingchenggensis*, an unusual two-component regulatory system was discovered at the end of a polyketide synthase-containing BGC that directs the production of a yellow pigment [51]. The so-called AtcK/AtcR two-component system differs from conventional kinase-response regulator pairs in that its two genes are not co-expressed in an operon but instead are oriented convergently (Figure 3C); how their expression is coordinated is not yet clear. The phosphorylated AtcR response regulator directly activates transcription of the adjacent *milR3* gene, which encodes a SARP (*Streptomyces* antibiotic regulatory protein) family regulator. MilR3, in turn, has differential effects on the expression of at least three BGCs, positively activating the expression of its associated yellow pigment-producing BGC, while simultaneously stimulating expression of the milbemycin BGC and repressing the expression of the nanchangmycin BGC [51] (Figure 3C).

The functional role of cluster-situated regulators has also recently been expanded to include regulator sequestration, with the discovery of a novel anti-repression mechanism involving the lysolipin I BGC in *Streptomyces auratus* [52]. This BGC encodes four cluster-situated regulators (LysR1–R4), with two belonging to the ‘transcriptional enhancement’ (TenA) family of regulators (Figure 3D). Mutational analysis of one of these TenA family-encoding genes (*lysR2*) suggested that it functioned as an activator of lysolipin I production; however, biochemical studies indicated that LysR2 was unable to bind DNA, raising questions about how it exerted its regulatory effects. Instead, it modulated the activity of the LysR1 transcriptional repressor by associating with it and promoting LysR1 dissociation from the DNA, thereby relieving cluster repression [52] (Figure 3D). This mechanism of anti-repression/cluster activation is reminiscent of sigma factor control, where sigma factors can be sequestered away from their target promoters by dedicated anti-sigma factors [53].

## New directions for pleiotropic regulators

In working to understand the factors that control BGC expression, the cluster-situated regulators are often the easiest to identify, given their immediate proximity to their BGC target genes. However, the majority of these cluster-situated regulators are themselves subject to multiple regulatory inputs. Nutrient-responsive regulators are frequently central—and conserved—players in this process, controlling specialized metabolism through their regulation of cluster-situated regulators, while simultaneously governing the expression of distinct primary metabolic pathways. Some of the most ubiquitous members of this pleiotropic regulatory group include GlnR (nitrogen-sensing), PhoP (phosphate-sensing), and DasR (glucosamine 6-P-sensing). DasR belongs to the metabolite-responsive GntR family of transcription factors and functions predominantly as a direct repressor of specialized metabolism, shutting down expression of cluster-situated regulators [54,55]. GlnR, in contrast, is an orphaned two-component response regulator (lacking a partner sensor kinase), and in addition to controlling nitrogen metabolism [56], it exerts both positive and negative control over specialized metabolism by binding within promoter regions of cluster-situated regulators [57]. PhoP is another response regulator, but unlike GlnR, it functions together with a dedicated sensor kinase (PhoR) to govern phosphate homeostasis [58] and is associated with the (often indirect) repression of specialized metabolism [59]. Many other two-component systems have been implicated in the control of specialized metabolism, including the AfsQ1/Q2 [60,61] and MtrA/B systems [62], which both act by directly controlling the expression of cluster-situated regulators. Like GlnR, AfsQ1/Q2 is responsive to nitrogen concentrations [61], while MtrA/B is both essential for viability and impacts morphological development [14,62]. Notably, GlnR, AfsQ1, and MtrA share many regulatory targets. The reason for this has become clear with the defining of their consensus binding sites, which share significant sequence similarity (GTnAC–n6–GnnAC) [63,64]. This suggests that these regulators may compete for binding and further highlights the efficiency of the bacterial genome, where a short DNA segment can function as a hub for integration of regulatory inputs from multiple transcription factors.

While there are economies of scale at play in the regulation of BGCs, the (generally) large size of BGCs as a whole means that they occupy significant space within the streptomycete chromosome (10–15% of the chromosome; e.g., [65]). Consequently, they are not only affected by the activity of cluster-situated and pleiotropic regulators, but are also susceptible to the effects of nucleoid-associated proteins, which function to compact and organize the chromosome while simultaneously influencing gene expression. Within the streptomycetes, and in the context of specialized metabolism, Lsr2 is the best characterized nucleoid-associated protein. This actinobacterial-specific protein functions as a metabolic gatekeeper, repressing the expression of many BGCs [66]. Unlike most other pleiotropic regulators, Lsr2 does not typically control the expression of cluster-situated regulators; instead, it binds to AT-rich regions within and adjacent to BGCs where it both polymerizes along the chromosome and forms bridges with adjacent chromosomal regions to shut down expression of the intervening sequences [66,67]. Recent work in *S. venezuelae* has revealed that the repressive effects of Lsr2 can be alleviated by the activity of cluster-situated activators [67]. Here, the cluster-situated regulator CmlR could recruit RNA polymerase to the chloramphenicol BGC, with the subsequent RNA polymerase-mediated transcription effectively clearing Lsr2 from the cluster DNA. It will be interesting to determine whether this ‘counter-silencing’ activity is a common feature of cluster-situated regulators.

## Containment and delivery of specialized metabolites

As detailed above, BGC regulators dictate where and when specialized metabolic genes are turned on, but understanding the containment of specialized metabolism, and whether and how the dissemination of these molecules is controlled, is an area of considerable interest. *Streptomyces* sporulation has long been associated with the release of volatile terpenes like geosmin and 2-methylisoborneol, which are under the control of sporulation-associated regulators [68]. It has recently been discovered that, in some instances, the synthesis of these molecules occurs within proteinaceous nanocompartments that are assembled in the cell. Many terpene synthases are co-expressed with a Type 2B encapsulin family protein [69]. Indeed, in the streptomycetes, the 2-methylisoborneol-associated encapsulin is known as EshA [70], and it oligomerizes to form an icosahedral-like compartment, within which the terpene synthase enzyme is loaded [71]. This physical sequestration of terpene production from the rest of the cytoplasm may function to protect the cargo enzymes and/or provide a means of controlling or improving pathway flux. Intriguingly, an additional family of encapsulin-like proteins is associated with polyketide synthase-encoding BGCs [69], suggesting that this type of spatial sequestration may be more commonly associated with specialized metabolism than has been previously recognized.

Specialized metabolites themselves can also be spatially constrained within membrane vesicles. These lipid-based compartments can be released from the tips of vegetative hyphae [72] and frequently contain specialized metabolites [73–76]. Interestingly, there can be specificity associated with vesicle loading: in *Streptomyces albus* S4, one antifungal compound is preferentially packaged over another [77], while in *Streptomyces* sp. Mg1, production of the linearamycin metabolites actively drives vesicle formation, and the packaging of these antibiotics into vesicles is thought to enhance their dispersal within their local environment [78,79]. Broadly speaking, these vesicles seem to provide a means of concentrating their cargo metabolites and facilitating their delivery to target sites/organisms [76]. How vesicle formation is coordinated with metabolite synthesis remains a fascinating question.

## Looking forward

Specialized metabolite production is a complex and nuanced process. Genetic, (bio)chemical, evolutionary, and ecological studies have all contributed to our foundational understanding of BGC control and molecule production. These have informed a multitude of successful approaches to stimulate the expression of lowly expressed and uncharacterized BGCs, including using small-molecule elicitors, isolating prospective producers from novel locations, diversifying growth media, providing competitive environments, and employing synthetic and other genetic approaches. There remains tremendous scope to continue expanding the bounds of possibility, e.g*.*, by exploring the metabolic potential of different cell types, growth stages, and containment strategies, in working to access the full metabolic potential of the streptomycetes.

Making sense of the multifaceted and complex regulation of BGCs represents both a challenge and an opportunity. There is increasing evidence that manipulating any one of the many regulators controlling a particular BGC can lead to cluster activation. An important prerequisite is knowing what regulators contribute to BGC control. Established techniques to map regulator binding sites, like chromatin immunoprecipitation coupled with deep sequencing (ChIP-seq), have been powerful tools in defining the regulatory landscape for individual regulators, while new approaches like *in vitro* protein occupancy display (IPOD) are being adapted for use in *Streptomyces* species and can provide unbiased insight into the sites of regulator binding within BGCs [80]. Databases like LogoMotif (https://logomotif.bioinformatics.nl/) are being developed to collate binding site information for actinobacterial transcription factors [81] and, importantly, are being integrated into BGC prediction programs like antiSMASH. These innovations will collectively allow for more informed application of existing genetic tools (e.g*.*, [66,82–89]) and will guide the development of new techniques and approaches.

## Summary

- Alternative cell types and low-density cultures can produce specialized metabolites that may not be seen during more conventional growth/culturing conditions.
- Beyond producing specialized metabolites in particular cell types, the biosynthetic enzymes for these compounds can be further sequestered within protein compartments in the cytoplasm.
- Packaging of metabolites into membrane vesicles appears to be a (relatively) general strategy for concentration and distribution.
- A complex interplay within and between cluster-situated regulators and external transcription factors governs BGC expression and allows for efficient integration of multiple regulatory inputs.
- Combining experimental tools aimed at characterizing regulator binding with databases like LogoMotif and antiSMASH that provide mechanisms of collating this information and applying the resulting knowledge to uncharacterized clusters, will advance our ability to rationally manipulate BGC expression.

## Open Access

Open access for this article was enabled by the participation of McMaster University in an all-inclusive Read & Publish agreement with Portland Press and the Biochemical Society under an agreement with OCUL.

## Abbreviations

BGCs: biosynthetic gene clusters
CDA: calcium-dependent antibiotic
IPOD: *in vivo* protein occupancy display
PKS: polyketide synthase
SARP: *Streptomyces* antibiotic regulatory protein
